# *Pseudomonas* PB1-Like Phages: Whole Genomes from Metagenomes Offer Insight into an Abundant Group of Bacteriophages

**DOI:** 10.3390/v10060331

**Published:** 2018-06-16

**Authors:** Siobhan C. Watkins, Emily Sible, Catherine Putonti

**Affiliations:** 1 Department of Biology, Loyola University Chicago, Chicago, IL 60660, USA; siv.watkins@nmt.edu (S.C.W.); esible@gradcenter.cuny.edu (E.S.); 2 Department of Computer Science, Loyola University Chicago, Chicago, IL 60660, USA; 3 Bioinformatics Program, Loyola University Chicago, Chicago, IL 60660, USA

**Keywords:** bacteriophage, Pbunaviruses, *Pseudomonas* phage PB1, uncultivated phage genomes

## Abstract

Despite the abundance, ubiquity and impact of environmental viruses, their inherent genomic plasticity and extreme diversity pose significant challenges for the examination of bacteriophages on Earth. Viral metagenomic studies have offered insight into broader aspects of phage ecology and repeatedly uncover genes to which we are currently unable to assign function. A combined effort of phage isolation and metagenomic survey of Chicago’s nearshore waters of Lake Michigan revealed the presence of Pbunaviruses, relatives of the *Pseudomonas* phage PB1. This prompted our expansive investigation of PB1-like phages. Genomic signatures of PB1-like phages and Pbunaviruses were identified, permitting the unambiguous distinction between the presence/absence of these phages in soils, freshwater and wastewater samples, as well as publicly available viral metagenomic datasets. This bioinformatic analysis led to the de novo assembly of nine novel PB1-like phage genomes from a metagenomic survey of samples collected from Lake Michigan. While this study finds that Pbunaviruses are abundant in various environments of Northern Illinois, genomic variation also exists to a considerable extent within individual communities.

## 1. Introduction

Research on bacterial viruses (bacteriophages) has progressed remarkably over the last 100 years [1], and phages are now regarded as ubiquitous engineers of bacterial community structure and metabolism [2,3,4,5]. Despite this, the quantity of phage-related information stored in biological sequence repositories is meager. To date there are less than 2000 distinct species represented in GenBank, which is largely a reflection of the many challenges of working with phages in the laboratory [6]. High-throughput sequencing platforms have been instrumental in advancing current understanding of microbial community diversity [7]. Viral metagenomic studies of phage communities have significantly improved our ability to examine and understand viral diversity in the wild [8,9,10]. Meta-analyses have expanded the viral catalogue postulating new branches of the evolutionary tree for viruses [11,12,13]. Furthermore, metagenomics has permitted a glimpse into viral biogeography (e.g., [11,14,15,16]; see review [17]).

Methods for molecular [18,19,20] and computational [21,22,23] assessment of viral communities are being continuously developed and refined. The aforementioned methods, as well as other approaches employed in studies (e.g., clustering contigs based upon nucleotide usage and/or coverage (see review [24]), have resulted in the generation of numerous complete or near complete genomes of uncultivated prokaryotes [25,26,27,28,29,30,31,32,33,34,35,36,37] and viruses [11,28,30,34,38,39,40]. Additionally, complete phage genomes have been assembled from viral metagenomes through manual curation and BLAST searches [11,15,33,41,42,43,44,45,46,47,48]. Given the advances in DNA sequencing technology throughput, high-quality genomes derived solely from metagenomes are anticipated to become commonplace, challenging the way in which we classify new viruses [49]. Nevertheless, with the recent assemblies of the largest phage genome to date [11] and the first genomes of freshwater *Actinobacteria*-infecting phages [48], metagenomics has greatly expanded our understanding of phage genomic diversity.

The study presented here was initially informed by our previous work, in which four *Pseudomonas aeruginosa*-infecting PB1-like phages (genus: *Pbunavirus*) were isolated from the nearshore waters of Lake Michigan [50]. Other members of this genus have been isolated from freshwater, sewage and soil and predominately infect *Pseudomonas* species [51]. While *Pseudomonas* phage PB1 is the type species for *Pbunavirus*, over 40 members of this genus have been suggested on the basis of homology assessments [52]. Currently there are 31 *Pbunavirus* species with RefSeq genomes publicly available. These dsDNA viruses have a genome ~66 Kbp in length with 88 to 97 predicted coding regions. Given its lack of a recognizable integrase, it is presumed to be obligately lytic [53]. Recently, PB1-related phages have been isolated from sewage samples in Germany [54], Poland [55], and Portugal [56], wastewater in France [57], and river water in Brazil [58].

While *Pseudomonas* is a truly multi-faceted microorganism and is generally regarded as having a high presence in the aquatic environment, it is not a dominant member of freshwater habitats [59,60], from which we previously isolated four PB1-like phages [50]. Nevertheless, Pbunaviruses have been found in a variety of ecological niches across the globe [51,54,55,56,57,58]. This parallels observations for several marine phages that have been found across large geographic distances [11,13,61]. This led us to hypothesize that Pbunaviruses are widespread in nature. Integrating molecular and computational methods, the presence and abundance of PB1-like phages was assessed in soil, freshwater, and sewage samples.

## 2. Materials and Methods

### 2.1. Samples

Samples were collected from three bodies of freshwater: (1) Lake Defiance (42°19′19.4″ N 88°13′37.5″ W) in Moraine Hills State Park (McHenry, IL, USA) is an isolated lake/bog; (2) Grass Lake/Fox River (42°26′46.4″ N 88°10′50.2″ W) in the Chain O’Lakes State Park (Spring Grove, IL, USA) is located within a large interconnected chain of lakes and waterways; and (3) Lake Michigan’s Hartigan Beach Park (42°00′06.3″ N 87°39′22.3″ W). Two biological replicates, each 4 L, were collected from Lake Defiance and Grass Lake in August 2015. Five temporal replicates, each 4 L, were collected from the Hartigan Beach site (June–August 2015). Additionally, four samples of activated sludge, in triplicate (50 mL) were collected from the aeration basins of O’Brien Water Reclamation Plant (Skokie, IL, USA) in October 2014. Four replicate soil samples (50 g) were collected from the Loyola Lakeshore campus in August 2015. Each sample of activated sludge and soil were diluted with 500 mL phosphate buffered saline (PBS), agitated in a shaking incubator overnight. Sample locations are shown in Figure S1.

### 2.2. Viral DNA Extraction

Virus-like particles were purified from the collected samples through successive filtration: initially, through a sterile 0.45 μm bottle-top cellulose acetate membrane filter (Corning Inc., Corning, NY, USA) to remove plant matter, sand, debris, and eukaryotic cells, then through a 0.22 μm polyethersulfone membrane filter (MO BIO Laboratories, Carlsbad, CA, USA) to remove bacterial cells. The filtrate was then filtered and concentrated using a 0.10 μm polypropylene filter (EMD Millipore Corp, Billerica, MA, USA) with the Labscale™ tangential flow filtration (TFF) system (EMD Millipore Corp, Billerica, MA, USA) according to the manufacturer’s instructions. DNA was extracted from the TFF fraction using the MO BIO Laboratories UltraClean^®^ DNA Isolation Kit (Carlsbad, CA, USA). The protocol recommended by the manufacturer was followed with the exception of an additional heat treatment at 70 °C for 20 min prior to initial vortexing.

### 2.3. PCR Amplification and Amplicon Analysis

PCR primers were designed to target conserved regions among the Pbunaviruses within the DNA of the collected samples. Primers and their expected amplicons (within the PB1 genome, GenBank: NC_011810) were queried against the nr/nt database. Five primer pairs were selected given their specificity for Pbunaviruses (Table 1). PCR primers were obtained from Eurofins MWG (Louisville, KY, USA). Thermal cycling conditions were designed based upon the individual primer pair’s T_m_ and expected amplicon size. PCR amplicons were purified (E.Z.N.A.^®^ Cycle Pure Kit, Omega Bio-tek, Norcross, GA, USA) and sequenced in both directions via Sanger sequencing (Genewiz, South Plainfield, NJ, USA). Consensus sequences were queried via BLASTn. Sequences were aligned in Geneious, and consensus sequences were assessed for phylogenetic relatedness, visualized via a neighbor-joining tree created with the Geneious Tree Builder tool (v 8.0.5, Biomatters Limited, Auckland, NZ) using the Jukes-Cantor distance model with bootstrap resampling (100 replicates).

### 2.4. Determination of the Presence of PB1 and Pbunaviruses in Publicly Available Metagenomic Datasets

Raw sequence reads (fasta or fastq format) for publicly available viral metagenomes from freshwater, hot springs, wastewater, and soil samples were collected from the SRA database. Table S1 lists the datasets. Additional marine viromes were downloaded from the iMicrobe project (data.imicrobe.us), and datasets collected from open sea sampling expeditions, including raw read data from the Pacific Ocean Virome study [62], the Global Ocean Sampling Expedition [63], the Broad Marine Phage Sequencing Project (https://www.broadinstitute.org/annotation/viral/phage/home.html), and the Ocean Viruses project [64]. For each individual sample, sequence reads were assembled using Velvet [65] with a hash size of 31. A local BLAST database was created for each dataset and again the PB1 amino acid sequences were compared via blastx.

The highest scoring hit (with respect to both sequence identity and query coverage, bitscore) for each PB1 gene and individual viral metagenome assemblage was next compared to the threshold of similarity for Pbunaviruses generated in our prior work [66]. Briefly, each PB1 gene was compared to all other *Pbunavirus* genomes and all non-Pbunavirus phage genomes via BLAST. Intragenus and intergenus sequence similarity scores (again assessed via the BLAST bitscore) were calculated to ascertain the “informativity” of a PB1 gene. PB1 genes exhibiting sequence similarity on par with or greater than that of its most distant *Pbunavirus* relative (BcepF1) were deemed “uninformative” as signatures for PB1 and Pbunavirus genomes. Each viral metagenome’s sequence similarity to PB1 informative genes are represented in the atlas as the average of the query coverage and sequence identity values.

### 2.5. Assembly and Annotation of Uncultivated PB1-Like Genomes

Each of the Chicago area Lake Michigan viral metagenomic datasets [67] was assembled individually using the Geneious assembler (Geneious (v 8.0.5), Biomatters Ltd., Auckland, NZ), the SPAdes assembler [68] using the “meta” flag for metagenomic samples, and the Velvet assembler [65]. Geneious assemblies repeatedly produced greater N50 scores and were selected for further analysis. Contigs of length ≥ 20 Kbp were queried against the complete nr/nt database via the BLAST web interface. Contigs returning high sequence similarity (e-value < 0.00001) to PB1 (GenBank: NC_011810) or other Pbunaviruses were investigated further. From this pool, each contig was then used as a “reference genome” to which the raw reads for individual viral metagenomic samples were mapped using Bowtie2 [69]. This additional second stage mapping process served to quantify genome coverage and in some cases extend contigs. At no time was the RefSeq PB1 genome or any other published *Pbunavirus* genome used to “fish” for reads: assembly to complete full genomes were generated a priori. Furthermore, in several cases the complete genome was produced during the initial assembly. All genomes were then manually inspected, and coverage was computed using BBMap (sourceforge.net/projects/bbmap/). Genomes from five viral metagenomic datasets were removed from further consideration as they contained regions (≥100 bp) of low coverage (<5 paired end reads). Annotations were generated, using Geneious, according to the most recent PB1 annotation (GenBank: NC_011810), and manually inspected again. Genome sequences were deposited in GenBank, Accession numbers KT372690 through KT272698.

### 2.6. Comparative Genomics

The nine uncultivated Lake Michigan PB1-like genome sequences were adjusted to the same frame as the PB1 RefSeq sequence. Genome alignments were performed using the progressive Mauve algorithm [70] and visualized in Geneious. These nine genomes were compared to other available *Pseudomonas*-infecting *Pbunavirus* genomes: phages LMA2 (GenBank: NC_011166), SN (GenBank: NC_011756), 14-1 (GenBank: NC_0111703), F8 (GenBank: NC_007810), PB1 (GenBank: NC_011810), LBL3 (GenBank: NC_011165), φVader (GenBank: KT254130), φMoody (GenBank: KT254131), φHabibi (GenBank: KT254132), and φFenriz (GenBank: KT254133). A neighbor-joining phylogenetic tree was created for the publicly available genomes, including the four Lake Michigan isolates [50], and the nine uncultivated PB1-like genomes based upon this alignment. This tree was created with the Geneious Tree Builder tool using the Jukes-Cantor distance model with bootstrap resampling (100 replicates).

## 3. Results

### 3.1. Hunting for Pbunaviruses in Soil, Freshwater and Activated Sludge

**Figure 1:**
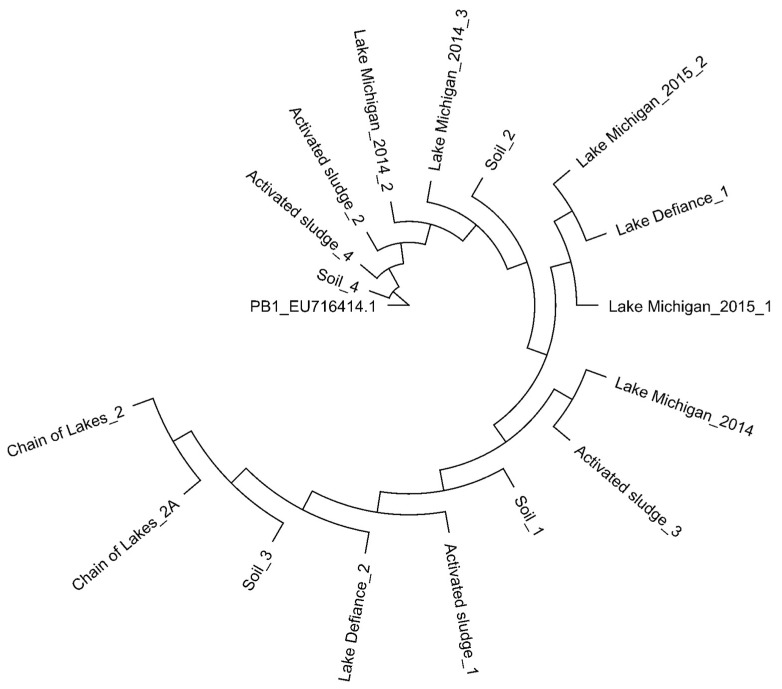
Phylogenetic comparison of PCR amplicons of the genes encoding hypothetical proteins PB1_gp84, 85, 86 generated from environmental DNA. Neighbor-joining tree was generated based on the Jukes-Cantor distance method.

In our previous work, we evaluated the coding sequences of PB1 and Pbunaviruses to identify genes (called “informative genes”) that can be used as signatures of PB1 strains as well as other Pbunaviruses [66]. We designed primer pairs targeting five genomic regions (see Methods; Table 1). These genomic regions target genes encoding hypothetical proteins as well as the annotated helicase (primer pair 3 in Table 1). Primer sequences as well as expected amplicon sequences (using the PB1 genome, GenBank: NC_011810) were queried using BLASTn; resulting hits were only to *Pbunavirus* species. Replicate samples of soil, activated sludge, and freshwater were collected from locations throughout northern Illinois (see Methods). Samples were filtered and concentrated, and the community DNA extracted and amplified with each of the primer sets (see Methods). DNA isolated from three Lake Michigan collections, made the year prior (2014) and within our existing collection, were amplified as well. Sanger sequencing of the resulting amplicons confirmed specific amplification of genes belonging to the Pbunaviruses; sequences were found to have a coverage >99% and sequence identity >99% to a *Pbunavirus* sequence in GenBank. Sequenced amplicons produced by primer pair 5 (amplifying genes encoding hypothetical proteins PB1_gp84, 85, 86) were using to construct a neighbor joining phylogenetic tree (Figure 1).

### 3.2. Searching for PB1 in Soil, Freshwater, and Activated Sludge Viral Metagenomes

**Figure 2:**
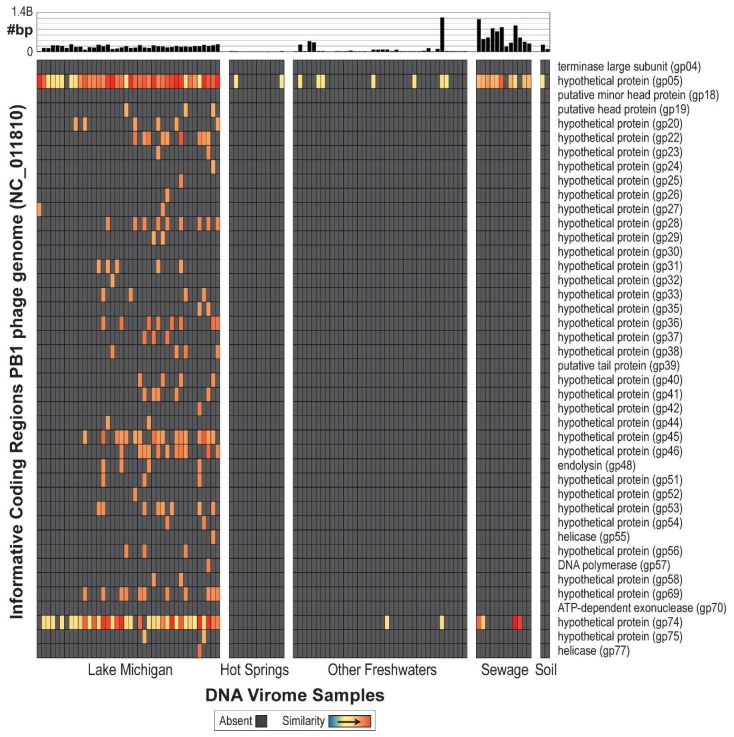
PB1 genome atlas, demonstrating quality hits to the PB1 genome across environmental viral metagenomes. Genes for which no hit was detected are indicated by grey. The bar graph at the top of the figure represents amount of data collected for that sample.

In total, 104 DNA viral metagenomes (from various environments) were inspected for the presence of PB1-like phages, including 40 from Lake Michigan nearshore waters (nine from collections made in 2013 [71] and 31 from 2014 [67]), 12 from hot springs, 38 from other freshwater sources, 12 from sewage, and two from soil (Table S1). Raw data was gathered for all data samples and assembled individually (see Methods). First, a purely BLAST-based approach was evaluated; all 104 viral metagenome samples produced high-quality hits to currently annotated PB1 genes (Figure S2). The 104 metagenomes were next re-evaluated considering only ORFs informative of PB1 genes listed in Figure 2. These genes are present within PB1 strains and do not exhibit sequence similarity to gene sequences within non-Pbunaviruses. As can be seen in Figure 2, many of metagenomes do not produce a signal (shown in gray) signifying that the viral metagenome did not include a sequence similar to the annotated gene sequences of PB1-like phages. Nevertheless, informative genes are detected within two hot spring samples, 47 freshwater samples, 11 sewage samples, and one soil sample. With the exception of the Lake Michigan samples, many of the samples identified only one informative gene. It is worth noting that comparable analyses were conducted for viral metagenome sets from marine environments, although no signal of the presence of PB1 was detected. To date, no *Pseudomonas*-infecting Pbunaviruses have been collected from marine/oceanic samples.

### 3.3. Unique PB1-Like Phage Genomes Can Be Assembled and Closed from Viral Metagenome Data Generated from Lake Michigan

Given the abundance of informative genes within the 31 Lake Michigan datasets collected during 2014 (Figure 2), each was analyzed to determine the presence of complete or near-complete PB1-like phage genomes. De novo assemblies were performed for these 31 paired-end sequence datasets [67], exploring numerous different assemblers and parameters (see Methods). High-quality contigs were then queried via BLAST for the presence of PB1 genes. Hits were identified within contigs for 14 of these data sets. Further examination revealed that nine contained single contigs representative of complete, high coverage (15 to 220×) genomes of PB1-like phages. The five other datasets were excluded because of low coverage. The nine genomes ranged in size from 65,762 to 66,283 bp in length, each comprising 85–89 annotated genes (Table 2). Overall, the nine genomes had high nucleotide sequence identity to each other—greater than 99% (Table S2). In addition, each genome was assembled from a different water sample, either collected on different dates, at different locations or found within a different biological replicate (Table 2). Assembly did not require a subsequent laboratory-based effort to extend or close the genomes; rather each genome was fully assembled based on metagenomic data alone.

**Figure 3:**
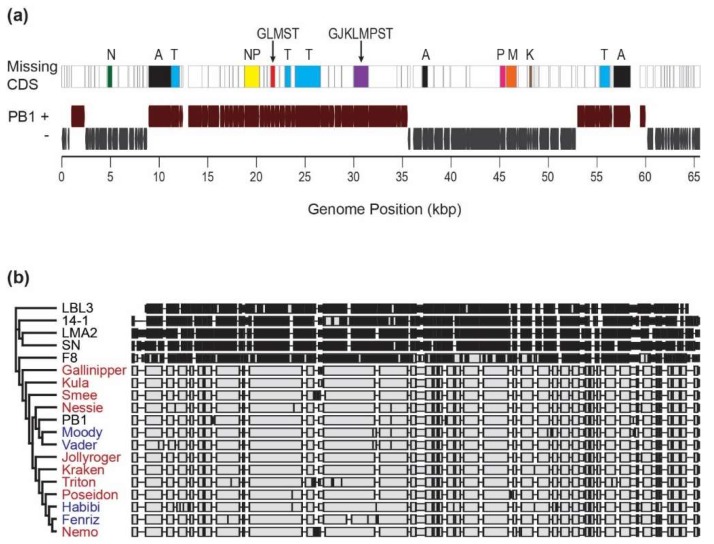
(**a**) Genome map of PB1-like genomes. CDS regions which are missing/disrupted in the new uncultivated viral genomes are indicated (A = All 9 genomes; G = Gallinipper; J = Jollyroger; K = Kraken; L = Kula; M = Nemo; N = Nessie; P = Poseidon; S = Smee; T = Triton). (**b**) Phylogenetic comparison of uncultivated Lake Michigan PB1-like viruses (red) to *Pseudomonas*-infecting Pbunaviruses. PB1 strains indicated in blue font are phages previously cultured and isolated from Lake Michigan [50].

Comparing the uncultured PB1-like genomes to the PB1 RefSeq genome record revealed collinear blocks common to all, as well as open reading frames (ORFs) (all homologous to annotated hypothetical proteins) which were absent throughout the entire cohort (Figure 3a). The ORFs absent from or disturbed (often by a frameshift mutation or large indel) from one or more of the uncultivated Lake Michigan PB1-like viruses can be found in Table S3. A phylogenetic comparison of the candidate phages against other *Pseudomonas*-infecting members of the Pbunaviruses and the genomes of four PB1-like phages previously isolated from Lake Michigan [50] was performed (Figure 3b). The nine uncultivated Lake Michigan PB1-like viral genomes demonstrated closest similarities to PB1 and the previously isolated Lake Michigan phages and exhibited marked differences from the genomes of the other Pbunaviruses.

## 4. Discussion

Given the ubiquity of *Pseudomonas*, it is not surprising that Pbunaviruses have been previously isolated from a vast array of environments [50,51,54,55,56,57,58]. By targeting a collection of species and genus specific genes, Pbunaviruses were detected within soil, freshwater, and activated sludge samples collected here. Comparison of these amplicon sequences revealed sequence diversity; contrary to our initial expectations, samples collected from the same geographical location or source (soil, freshwater, activated sludge) did not clade together. While the amplicon sequences indicate that Pbunaviruses (or very close relatives) are present in northern Illinois, the phylogenetic tree suggests genetic variation amongst the samples that is not dictated by the type of environment sampled. Comprehensive analysis determined that the PB1-like phages, are, in fact, very well represented throughout datasets collected from Lake Michigan (Figure 2). The samples sequenced from collections made in the summer of 2014 [67] included more “informative genes” than the sequences from the samples collected in the summer of 2013. Nevertheless, these are the same sites from which the PB1-like phages φVader, φMoody, φHabibi, and φFenriz were isolated during the summer of 2013 [50].

The evidence for the presence of Pbunaviruses in other freshwaters, soils, wastewater, and hot springs metagenomes, is however, tenuous (Figure 2). This may be a result of differences in the sample sites themselves (e.g., oligotrophy, season, depth, etc.), sample preparation, sequencing depth and/or technology, or PB1 abundance within the particular sample. Interestingly, PB1 gp05 (128 amino acids in length) and gp74 (150 amino acids in length), both annotated as hypothetical proteins, are detected—often at very high levels—within these samples. Both of these genes were found to be *Pbunavirus*-specific, not present in any other sequenced phage genome. Furthermore, each was queried (tBLASTn) against the complete nr/nt database; all hits returned were to Pbunaviruses. BLASTp analysis confirmed that gp05 is exclusively found with *Pbunavirus* sequences and PB1′s gp74 amino acid sequence exhibited only modest sequence homology (57% query coverage and 42% sequence identity) to a hypothetical protein within the T4-like *Pseudomonas putida*-infecting phage pf16 [72]. These proteins are of particular interest with regards to characterizing their function. The detection of these two hypothetical proteins in other viral metagenomic samples can be the result of: (1) the presence of distant *Pbunavirus* relatives; (2) evidence of prior gene sharing between Pbunaviruses and other viral species; or (3) these similar sequences originating independently.

During examination of a viral metagenomic survey of Lake Michigan, we were able to assemble nine separate PB1-like genomes, de novo from the data generated. Variation was observed between the uncultivated assembled viruses including size. Three of the genomes, Kraken, Kula and Nemo, came from biological replicates collected from the same site (Calumet Beach) on 5 August 2014. From the pairwise assembly of the genomes of Kula and Nemo, 529 nucleotide differences were observed (Table S2). This suggests that even within a niche (as replicates were collected within a 5 m area [67]), significant viral microdiversity exists. Interestingly, a complete PB1-like genome was unable to be assembled from the fourth biological replicate collected. Variation was also observed among the nine genomes with respect to the number of ORFs identified. It is important to note that reading frames and predicted functions were determined based upon the PB1 genome annotation. Regions which are missing/disrupted in the new uncultivated viral genomes were predominantly PB1 annotated hypothetical proteins (Figure 3A; Table S3). Nevertheless, disruptions were observed in all 9 genomes for the minor head protein (PB1 gp18) and helicase (PB1 gp77). Nevertheless, the genomes of all nine uncultivated viruses were more similar to PB1-like viruses than other members of the genus.

Significant sequence coverage suggests that these phages were present in the Lake Michigan samples in high abundance. The nine genomes essentially fell out of the data, providing a more genuine representation of viral abundance than the number of hits alone. Luo et al. [73] estimated genomes may be assembled most accurately from metagenomes with a coverage of 20×—the coverage of many of the genomes examined in this study far exceeded this measure (Table 2). As with the discovery of crAssphage, ubiquitous in human fecal metagenomes [39], ultimately the efficacy of assembling entire genomes from complex environmental datasets is reliant on the initial high abundance of the organism, or an alternative factor which effectively places it as the low hanging fruit in that data. However, as generating complete genomes of other characterized phage genomes from our data was not possible, the former is likely the case here.

We were unable to produce a complete genome assembly of a PB1-like virus from the nine viral metagenomic dataset from Lake Michigan collected the year before and hits to informative genes in the PB1 genome were low throughout (Figure 2). This suggests that the incidence of PB1-like viruses was considerably higher, for whatever reason, during the 2014 sampling effort, and that the presence of this group of viruses is likely to be, unsurprisingly, dynamic. Nevertheless, we are confident that PB1 viruses were present during 2013 as four were isolated and characterized from these very same samples [50]. The results of culture-independent and culture-dependent methods further supports the presence of a dynamic community.

Although culture-based studies of phages can provide multiple key aspects of a phage’s ecology, the many challenges with working with phages in the laboratory [6] limit our understanding of their genetic diversity. Viral metagenomic studies, however, have the potential to uncover far more than just a phage’s genome [21]. Through comparative viral metagenomics we were able to identify samples for which PB1-like viruses were present as well as abundant, thereby providing insight into the phage’s ecology. Hypothesis-driven data mining coupled with experimental work, such as the approach presented here for the investigation of PB1-like viruses, has significant potential to greatly expand our understanding of phage ecology.

## Figures and Tables

**Table 1:** PCR primers used in this study. Expectation of amplification determined by querying the primer sequences via BLASTn to nr/nt database. HP = hypothetical protein.

Primer Pair	Forward Primer (5′–3′)	Reverse Primer (3′–5′)	Annotated Coding Sequence (CDS) in Amplicon	Strains/Species Expected to Amplify
1	CTACGGCCGTGCAGAC	CTCCATGTGTGGCATCC	PB1 gp10 (HP), PB1 gp11 (HP)	SPM-1, PB1, F8
2	ACCTTCTTCGGCATCCTC	TGTGGTCACCGTATTCCA	PB1 gp23 (HP), PB1 gp24 (HP)	DL60, DL52, vB_PaeM_C1-14_Ab28, SPM-1, PB1, F8
3	CGCCATAATAGGCTCCAA	CGAGACATTCGCTGATGA	PB1 gp55 (helicase)	SPM-1, PB1, F8
4	ACCGACTCACGACGATGG	CGGCAAGGTGTTCGCTTA	PB1 gp68 (HP), PB1 gp69 (HP)	PB1
5	CGTCGAGGATGCTGATGG	GGCAGGTCCGAAGGCTAC	PB1 gp84 (HP), PB1 gp85 (HP), PB1 gp86 (HP)	vB_PaeM_LS1, vB_PaeM_E215, KPP22M1, vB_PaeM_E217, NP3, phiKTN6, KPP22M3, KPP22M2, KP22, vB_Pae_PS44, LMA2, vB_PaeM_CEB_DP1, vB_PaeM_PAO1_Ab29, vB_PaeM_PAO1_Ab27, KPP12, NH-4, PB1

**Table 2:** Descriptive information for the nine uncultivated PB1-like genomes from Lake Michigan samples.

Phage	Isolation (Beach/Date)	Coverage (Average)	Length (bp)	# Annotated Genes	Accession Number	SRA Accession (Raw Reads)
Gallinipper	95th Street/10 June 2014	30	65,917	89	KT372690	SRX956318
Jollyroger	57th Street/8 July 2014	200	65,795	90	KT372691	SRX995828
Kraken	95th Street/5 August 2014 (Biological Replicate 1)	15	65,762	88	KT372692	SRX995836
Kula	95th Street/5 August 2014 (Biological Replicate 2)	20	65,762	89	KT372693	SRX995836
Nemo	95th Street/5 August 2014 (Biological Replicate 3)	20	66,283	88	KT372694	SRX995836
Nessie	Wilmette/13 May 2014	220	65,762	89	KT372695	SRX995816
Poseidon	Wilmette/5 August 2014	130	65,762	88	KT372696	SRX995833
Smee	Montrose/8 July 2014	115	66,278	89	KT372697	SRX995827
Triton	57th/5 August 2014	125	65,762	85	KT372698	SRX995835

## Supplementary Materials

The following are available online at http://www.mdpi.com/1999-4915/10/6/331/s1, Figure S1: Map of collection sites for this study. Figure S2: PB1 BLAST genome atlas. Bitscore values of BLAST queries for each PB1 gene in each metagenome is indicated. Table S1: Information about the viral metagenomic data sets evaluated for the presence of *Pseudomonas* phage PB1. Table S2: Sequence similarity between the nine uncultivated genomes assembled in this study. Table S3: List of coding sequences missing or disrupted genes in the nine uncultivated genomes.
